# Aerogels of Polypyrrole/Tannic Acid with Nanofibrillated Cellulose for the Removal of Hexavalent Chromium Ions

**DOI:** 10.3390/gels10070415

**Published:** 2024-06-22

**Authors:** Islam M. Minisy, Oumayma Taboubi, Jiřina Hromádková, Patrycja Bober

**Affiliations:** Institute of Macromolecular Chemistry, Czech Academy of Sciences, 162 00 Prague, Czech Republic

**Keywords:** polypyrrole, tannic acid, aerogels, adsorption, hexavalent chromium ions

## Abstract

The preparation of conducting polymer aerogels is an effective strategy to produce innovative materials with enhanced physicochemical properties. Herein, polypyrrole (PPy) aerogels were oxidatively prepared in the presence of tannic acid (TA) with different concentrations (2.5, 5, and 10% mole ratio to pyrrole monomer) under freezing conditions. Nanofibrillated cellulose (NFC) was added during the PPy/TA synthesis to enhance mechanical stability. The effect of TA concentration on the aerogels’ morphology, conductivity, thermal stability, and adsorption capacity was investigated. The conductivity of 9.6 ± 1.7 S cm^−1^ was achieved for PPy/TA prepared with 2.5% TA, which decreased to 0.07 ± 0.01 S cm^−1^ when 10% TA was used. PPy/TA aerogels have shown high efficacy in removing Cr(VI) ions from aqueous solutions. Adsorption experiments revealed that all the aerogels follow pseudo-second-order kinetics. PPy/TA prepared with NFC has a maximum adsorption capacity of 549.5 mg g^−1^.

## 1. Introduction

Hexavalent chromium (Cr(VI)) is classified as one of the top-priority toxic contaminants by the US Environmental Protection Agency, due to its high solubility, carcinogenicity, and oxidizing properties [1]. It can be generated by industrial activities of chromium electroplating, leather tanning, paint pigments, steel production, etc. [2]. Several techniques have been designed for remediation of heavy metals (including Cr(VI) ions) in wastewater. These techniques can be categorized into adsorption, reduction/oxidation, ion exchange, membrane separation technologies, solvent extraction, chemical precipitation/co-precipitation, bioremediation methods, etc. [3,4]. Unlike Cr(VI), Cr(III) is an essential dietary nutrient, which is 1000 times less toxic than Cr(VI) and can be easily precipitated at neutral conditions [5]. The most effective approaches for the remediation of Cr(VI) ions in water are selective adsorption [6] or catalytic reduction to Cr(III) ions. The ordinary chemical reduction of Cr(VI) consumes large quantities of reducing agents (e.g., iron(II) sulfate, NaBH_4_) and leads to the formation of byproducts that are difficult to recycle [7,8].

**Scheme 1:**
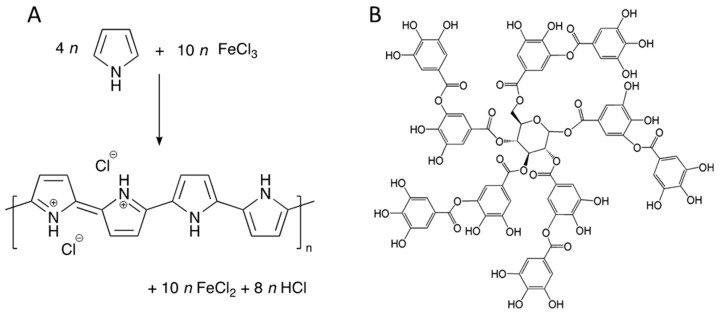
Oxidative polymerization of pyrrole with iron(III) chloride to PPy hydrochloride (**A**); the chemical structure of TA (**B**).

Various organic and inorganic adsorbents have been used to decontaminate wastewater containing Cr(VI) [4,9]. Among them, conducting polymer-based adsorbents have shown interesting simultaneous adsorption/reduction properties [10,11,12,13]. Conducting polymers are environmentally stable materials that can be prepared in various composite forms. Polypyrrole (PPy) is one of the most studied conducting polymers that can be easily prepared by the oxidative polymerization of pyrrole with iron(III) chloride as an oxidant (Scheme 1A). PPy is usually obtained as a powder with a relatively low specific surface area and poor mechanical properties [14]. The preparation of various PPy composites has been widely utilized to enhance the adsorption performance and catalytic reduction properties [10,15,16]. The preparation of conducting polymer aerogels is an effective strategy for the enhancement of their adsorption capacity. Recently, PPy aerogels with various organic stabilizers have been utilized for the adsorption of Cr(VI) ions from aqueous solutions, with a maximum adsorption capacity up to 497.5 mg g^−1^ [17]. Aerogels of PPy/nanofibrillated cellulose (NFC) were found to enhance the adsorption capacity of Cr(VI), although NFC has no affinity towards Cr(VI) ions [11].

Tannic acid (TA) is a natural water-soluble polyphenolic compound (Scheme 1B), which can be extracted from bark, fruits, leaves, and seeds of many plants, e.g., Caesalpinia spinosa, Rhus semialata, etc. [18]. It has a high affinity for metal ions, the ability to form complexes, and good electron transfer activity. This is due to its molecular structure of adjacent carboxyl and hydroxyl groups. In addition, it has interesting antioxidation, anti-inflammatory, and antiviral properties [19]. Therefore, TA is a promising candidate for removing heavy metal ions from wastewater. TA was found to reduce Cr(VI) to Cr(III), and form complexes of oxidized TA-Cr(III), which can be later separated by precipitation at neutral pH [20]. However, the high water solubility of TA limits its application in this field. This can be overcome by TA immobilization onto various insoluble substrates to improve the adsorption capacity and catalytic performance towards heavy metal ions, and facilitate the separation of the adsorbent from solutions. Recently, TA-functionalized microcrystalline cellulose was prepared through radiation-induced grafting polymerization [21]. This composite showed selective removal and recovery of Ga(III) and In(III) heavy metal ions from aqueous solutions, with a maximum adsorption capacity of 26.55 for Ga(III) and 35.63 mg g^−1^ for In(III) at pH 3 [21]. PPy/TA composites were prepared through oxidative polymerization of pyrrole with ammonium persulfate to improve the electrochemical performance of PPy [22]. Soft, conductive, porous and biocompatible PPy/TA hydrogels were obtained with iron(III) chloride as an oxidant and were used as a spinal cord scaffold [23]. PPy/TA/iron nanocomposite was used for the removal of Acid Yellow 42 with the Langmuir maximum adsorption capacity of 116.5, 119.1, and 140.1 mg g^−1^ at 40, 50, and 60 °C, respectively [24]. However, PPy/TA aerogels have not been tested before as adsorbents of Cr(VI) ions.

Herein, PPy/TA aerogels were prepared through cryopolymerization of pyrrole in the presence of different concentrations of TA. NFC was added to the polymerization medium of PPy/TA to improve the mechanical properties of the aerogels. The batch adsorption experiments of Cr(VI) onto various PPy/TA and PPy/NFC/TA aerogels were carried out as a function of time and concentration of Cr(VI) ions.

## 2. Results and Discussion

### 2.1. Characterization

#### 2.1.1. Morphology

**Figure 1:**
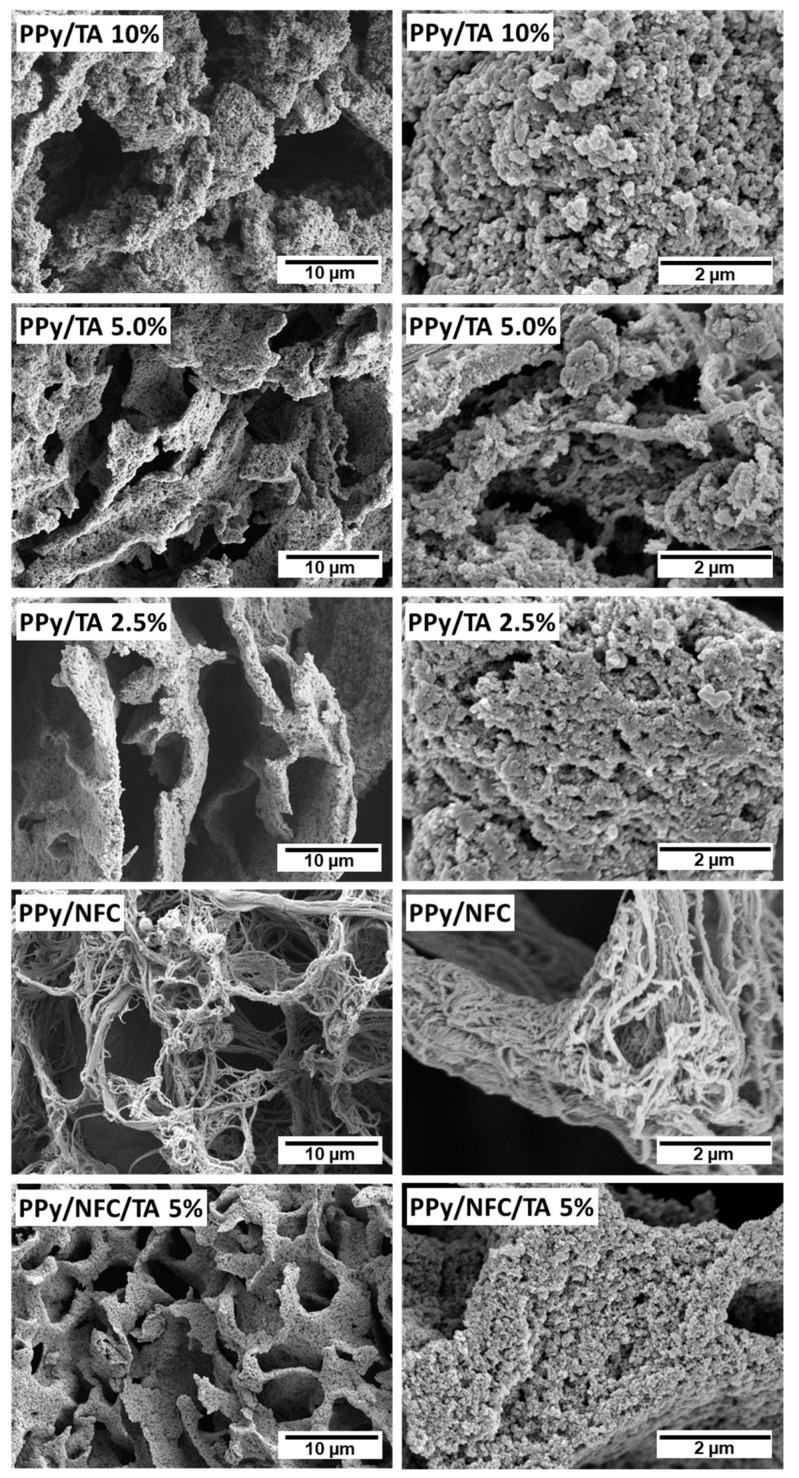
SEM micrographs of PPy/TA aerogels with 2.5, 5.0, and 10 mole% of TA, PPy/NFC aerogel, and PPy/NFC/TA 5% aerogel, at two different magnifications.

The surface morphologies examination reveals the formation of highly porous PPy/TA aerogels with different TA concentrations (Figure 1). The higher magnification shows that PPy/TA aerogel walls are composed of aggregates of spherical nanoparticles, which are much smaller than the particles of neat PPy, as shown in Figure 1 and Figure S1. In the case of PPy/NFC aerogel prepared in the absence of TA, PPy was fully deposited onto the NFC fibers’ surface, which works as the skeleton to form a 3D network, and no globular PPy particles were observed [11].

Similarly, a highly interconnected porous structure with sponge-like morphology was formed in the case of PPy/TA prepared in the presence of NFC (Figure 1). The aerogel of PPy/NFC/TA 5% has thicker walls compared with PPy/NFC due to accumulated deposition of PPy/TA particles onto it (as seen under the higher magnification). The hydrogen bonding between –N–H groups in PPy and the hydroxyl groups of TA and NFC secure the homogenous interaction between the aerogel components.

#### 2.1.2. FTIR Spectroscopy

**Figure 2:**
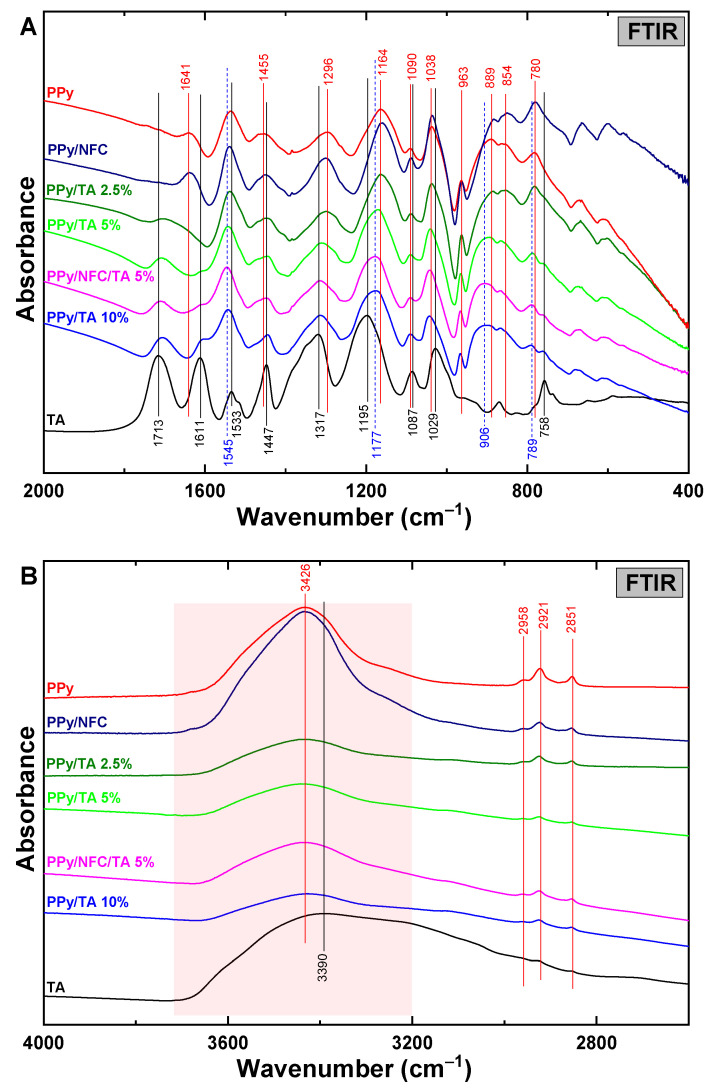
Infrared spectra of PPy, PPy/TA composites prepared with 2.5, 5.0, and 10 mole% of TA, PPy/NFC and PPy/NFC/TA 5% aerogels in the region of 450–2000 cm^−1^ (**A**) and 2600–3800 cm^−1^ (**B**).

The characteristic bands for TA are displayed in Figure 2. The intense peaks at 1713, 1611, 1317, 1195, 1087, and 1029 cm^−1^ represent C=O stretching, aryl C=C stretching from the aromatic ring, O–H deformation and C–O stretching of phenols, aryl oxygen stretching vibrations, and C-H bending vibration, respectively [25,26]. The peak at 1447 cm^−1^ is attributed to stretching vibration of –C–C– aromatic groups. The band at 758 cm^−1^ correspond to the C–H out-of-plane deformation.

The typical absorption bands of PPy at around 1533 and 1455 cm^−1^ correspond to the C=C and C–N stretching vibration in the pyrrole ring, respectively. The band at 1641 cm^−1^ can be related to the overoxidation of PPy. The band at 1296 cm^−1^ corresponds to C−H or C–N in-plane deformation modes. The breathing vibration of the pyrrole ring is given at 1164 cm^−1^. The bands 1090 and 1038 cm^−1^ belong to N–H^+^ deformation vibrations and C–H and N–H in-plane deformation vibrations, respectively. The peak at 963 cm^−1^ is attributed to the C–H out-of-plane deformation vibrations. The band at 854 cm^−1^ is attributed to C–H in-plane deformation and the bands located at 889 and 780 cm^−1^ are assigned to C–H out-of-plane ring deformation [27].

The FTIR spectra of PPy/TA (with various TA mole%), PPy/NFC, and PPy/NFC/TA aerogels are identical to the spectrum of neat PPy. However, the bands at 1611 and 758 cm^−1^, belonging to TA, attributed to aromatic C–O symmetric stretching vibration and C–H out-of-plane ring deformation, respectively, appear as small shoulders in the spectra of PPy/TA 10%. In addition, some bands exhibit shifts to higher wavenumbers, for instance, the bands at 1296, 1164, 889, and 780 cm^−1^ are blue-shifted toward 1317, 1177, 906, and 789 cm^−1^, respectively. It is worth noting that the characteristic absorption band of PPy at 1533 cm^−1^ is also shifted to 1545 cm^−1^ after adding at least 5% of TA into the PPy. This shifting may be caused by the interaction between the TA and PPy backbone, such as π–π stacking or/and electrostatic interactions.

In the region 3700 to 3000 cm^−1^, the spectrum of TA presents a wide and strong band centered at 3390 cm^−1^, and is assigned to the hydroxyl group (OH) stretching vibrations. The peaks 2921 and 2851 cm^−1^ are associated with the symmetric and antisymmetric –CH– stretching vibrations of the CH_2_ and CH_3_ groups, respectively [28]. The band at 3426 cm^−1^ observed in the PPy spectrum refers to the N–H stretching vibration in PPy becoming wider in the presence of TA. This can suggest the hydrogen bonding between PPy and TA [22].

#### 2.1.3. Raman Spectroscopy

**Figure 3:**
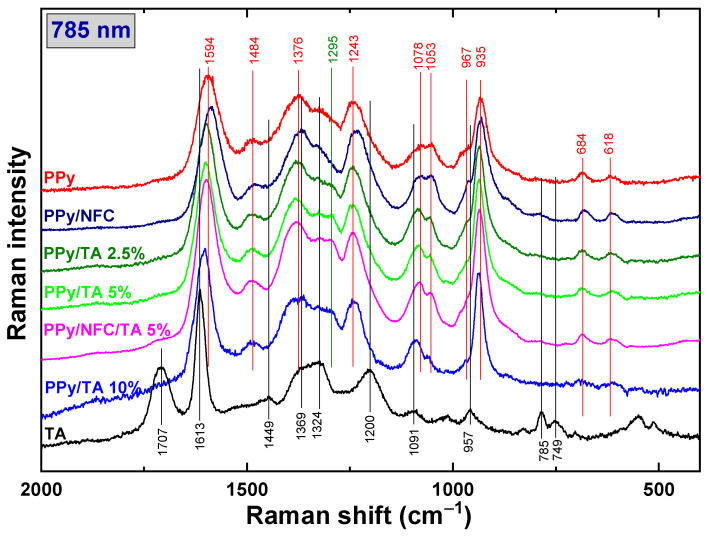
Raman spectra of PPy, PPy/TA aerogels prepared with 2.5, 5.0, and 10 mole% of TA, PPy/NFC and PPy/NFC/TA 5% aerogels.

Figure 3 displays the Raman spectrum of TA in which a broad band at 1707 cm^−1^ assigned to the stretching (C=O) vibration in the ester group is well-distinguishable. The band broadening is due to the large number of carbonyl groups present in TA, as well as the existence of various intra- and intermolecular hydrogen bonds [29]. The ring stretching vibrations in polyphenols are very prominent and highly characteristic of the aromatic rings (this band is observed at 1613 cm^−1^) [30]. In addition, other typical bands of TA are observed at 1449 cm^−1^ (ring stretching, OH in-plane bending), 1369 and 1324 cm^−1^ (CH deformation, C–OH deformation, ring stretching), 1200 cm^−1^ (CH in-plane bending, OH deformation), 1091 cm^−1^ (CH in-plane deformation), 957 cm^−1^ (C–COOH stretching), and 785 and 749 cm^−1^ (out-of-plane bending of –CH and –OH).

The bands in the spectrum of neat PPy are assigned as follows: C=C stretching in polaron structure at about 1594 cm^−1^, the skeletal, or C−C and C−N stretching vibrations are given at 1484 cm^−1^, ring stretching vibration at 1376 and 1324 cm^−1^ in bipolaron and polaron structures, respectively. The band at 1243 cm^−1^ is associated with the antisymmetrical C−H in-plane bending vibrations. The bands at 1053 and 1078 cm^−1^ are assigned to the C–H deformation in polaron and bipolaron structures, respectively. The bands at 967 and 935 cm^−1^ are attributed to the ring deformation vibrations of neutral PPy in neutral and bipolaron states, respectively [31,32].

Raman spectra of PPy/TA composites reveal the vanishing of the strong band at 1707 cm^−1^ (of TA) due to the complexation between TA and iron metal ions coming from the oxidant (iron(III) chloride) [30]. The intensity of the polaron band at 1053 cm^−1^ becomes less intense than the intensity of the bipolaron band (1078 cm^−1^) by increasing TA content. The same observation is demonstrated in the spectrum of PPy/NFC/TA 5%, where a decrease in the polaron band at the expense of the bipolaron band is observed that might be assigned to the better chain organization of the PPy in the presence of TA.

#### 2.1.4. Electrical Conductivity

The DC conductivity of pressed pellets of different aerogels shows that conductivity decreases by increasing the TA concentration, as presented in Table 1. The lowest conductivity of 0.07 S cm^−1^ was obtained for PPy/TA prepared with 10 mole% TA, and the highest of 9.6 S cm^−1^ was obtained for PPy/TA aerogel prepared with 2.5 mole% TA. The conductivity of neat PPy prepared at room temperature is usually around unit S cm^−1^, but increased to 23.7 S cm^−1^ when prepared under frozen conditions (−24 °C) [33,34]. The conductivity of PPy–NFC aerogel dropped by two orders of magnitude from 31 S cm^−1^ to 0.37 S cm^−1^ when prepared in the presence of TA (5 mole %) [11]. Many factors may lead to the conductivity decrease upon incorporating TA with PPy. The interaction between PPy and TA controls the morphology, which affects the conductivity. Also, due to its insulating nature, TA creates a physical barrier that blocks the charge transport pathways [22,35]. As the concentration of TA increases, the DC conductivity decreases. On the other hand, previous reports show that the addition of TA may lead to an enhancement in the ionic conductivity of PPy hydrogels (from 0.05 to 0.18 S cm^−1^ by increasing the TA from 0.15 wt% to 0.6 wt% then decreasing to 0.12 S cm^−1^ at 2.5% TA) [23].

#### 2.1.5. Thermal Stability

**Figure 4:**
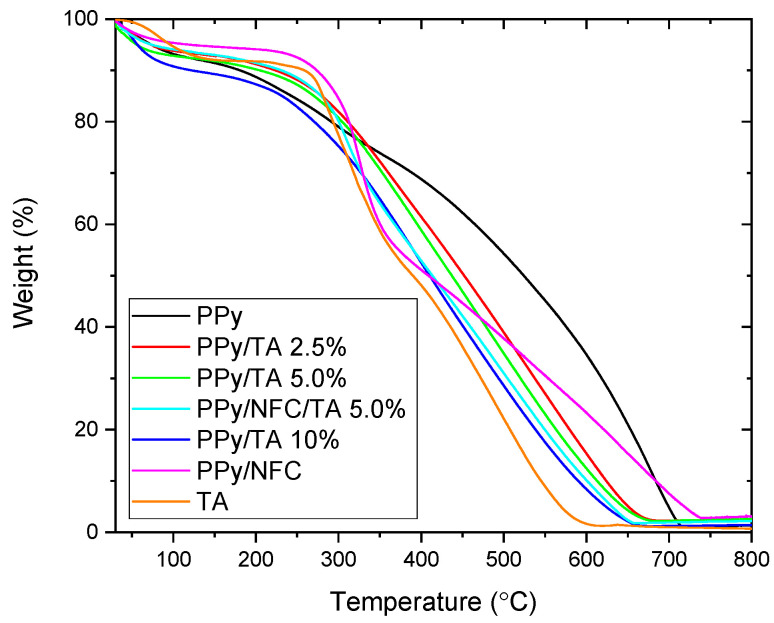
Thermal gravimetric analysis of neat PPy and TA powders, PPy/TA with various mole% of TA, PPy/NFC and PPy/TA/NFC aerogels.

The thermal gravimetric analysis of the various PPy aerogels shows that their thermal stability was slightly decreased by increasing TA content (Figure 4). Among the aerogels, PPy/NFC aerogel showed the highest thermal stability. For all the aerogels, the first gradual weight loss up to 110 °C is due to the loss of absorbed moisture. The second weight loss step at around 220 °C is assigned to the degradation of TA and cellulose macromolecules, and the complete loss of PPy dopant. The full decomposition of the aerogels took place around 680 °C in the case of PPy/TA 10%, 650 °C in the case of PPy/TA 2.5% or PPy/NFC/TA 5%, and 735 °C in the case of PPy/NFC aerogels. The neat TA and PPy powders fully decomposed at around 600 and 715 °C, respectively.

### 2.2. Adsorptive Removal of Cr(VI) Ions from Aqueous Solutions

It is well-known that pH plays a crucial role in adsorption by controlling the surface charge of the adsorbent and the adsorbate. PPy and its composites have always shown the highest adsorption efficiency towards Cr(VI) at acidic conditions (pH 2–3). Acidic conditions facilitate the electrostatic interactions and ion exchange between positively charged PPy chains and the predominant HCrO_4_^−^ anionic species [36].

**Figure 5:**
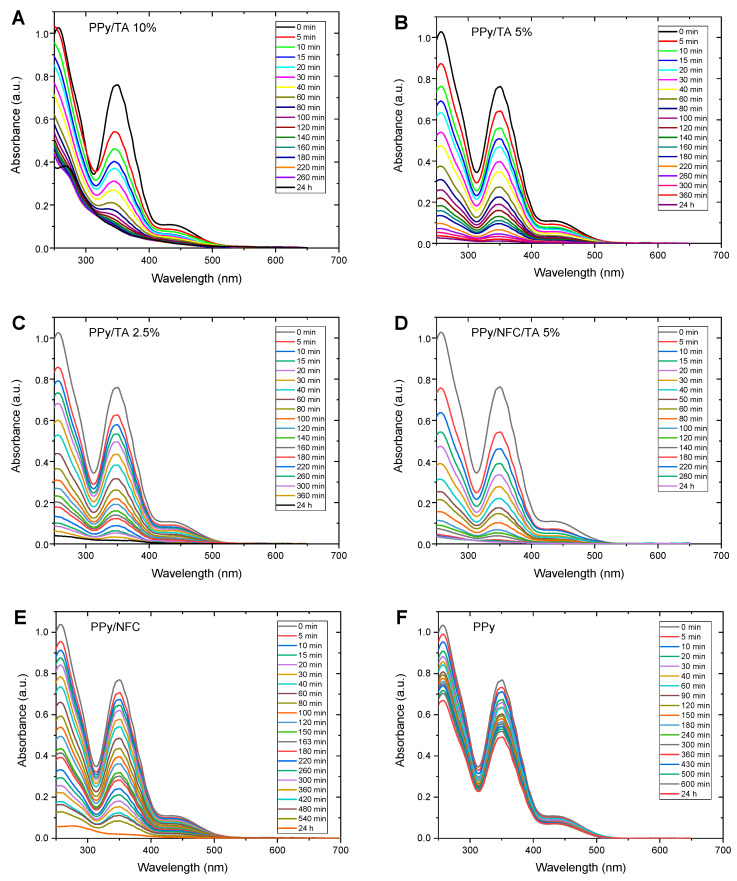
UV-visible spectra of Cr(VI) over time while adsorbing onto PPy/TA with 10 (**A**), 5.0 (**B**), 2.5 (**C**) mole% of TA, PPy/NFC/TA 5% (**D**), PPy/NFC aerogels (**E**), and neat PPy powder (**F**).

Herein, the efficiency of PPy/TA and PPy/NFC aerogels as adsorbents for removing Cr(VI) ions from aqueous solutions was studied at pH 2. Figure 5 presents the UV-visible spectra of Cr(VI) solutions followed over time when they came into contact with different PPy aerogels or neat PPy. In the case of PPy/TA 10% (aerogel with the highest content of TA), Cr(VI) peak at 350 nm disappeared the fastest compared with other adsorbents. However, the new peak at 272 nm and the high baseline observed at the end of the adsorption process were found as a result of the leaching of TA from the composite and complexes with Cr ions in the solution, respectively (Figure S2). Due to this reason, PPy/TA 10% was not further studied as an adsorbent. PPy composites prepared with 5 and 2.5 mole% of TA and in the presence of NFC showed much higher efficiency in the adsorption of Cr(VI) compared with neat PPy or PPy/NFC. In the case of PPy/TA aerogels with less than 10% TA, no TA release was detected spectrophotometrically, as proven in Figure S2. Neat PPy has a much lower affinity for Cr(VI) than the aerogels, while the neat NFC shows no affinity at all for Cr(VI) ions, and the Cr(VI) absorption spectra have not changed after 24 h (Figure 5 and Figure S3).

**Figure 6:**
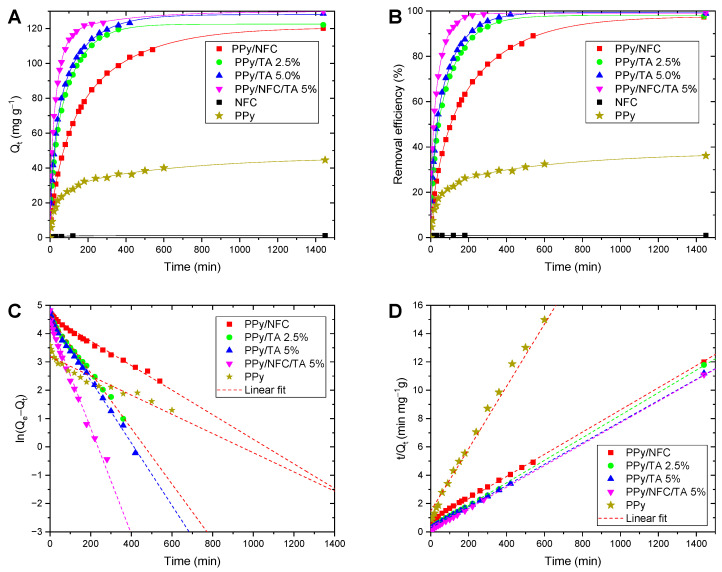
Time-dependent adsorption capacity (**A**) and removal efficiency (**B**) of Cr(VI) ions adsorbing onto PPy/TA with various mole% of TA, PPy/NFC, PPy/NFC/TA 5% aerogels, neat NFC and neat PPy powder. Pseudo-first-order (**C**), and pseudo-second-order (**D**) kinetic modeling.

Figure 6 shows the effect of contact time on the adsorption capacity of the prepared adsorbents and their efficiency in Cr(VI) uptake. In all the cases, the amount of Cr(VI) ions adsorbed onto the PPy aerogels was found to increase with time, and the adsorption rates were found to be faster during the initial adsorption stage and slowed down gradually with time until reaching the equilibrium state. This can be explained on the basis that at the beginning the adsorption takes place mainly on the external surfaces of the composites. After the saturation of the binding active sites on the surface, Cr(VI) ions gradually diffuse into the interior and the adsorption rate decreases.

The results indicate a great enhancement of the adsorption capacity and removal efficiency of the Cr(VI) ions by introducing TA to PPy, and more by adding NFC; however, NFC itself has shown no affinity for Cr(VI). Neat PPy powder has a low adsorption capacity and removal efficiency of only 36.2% after 24 h. PPy/NFC/TA 5% showed the highest removal rate, with 97.1% removal efficiency achieved after 3 h, and 99.4 after 24 h, compared with 63% removal efficiency (after 3 h) for PPy/NFC prepared in the absence of TA. TA possesses abundant carboxyl and phenol hydroxyl groups, which are protonated and positively charged in the acidic medium [37]. This can enhance the Cr(VI) adsorption by favorable electrostatic attraction of negatively charged Cr(VI) anionic species (Figure S4). In addition, the phenolic hydroxyl groups of TA can partially reduce Cr(VI) under acidic conditions to Cr(III) [20].

### 2.3. Adsorption Kinetics

The adsorption kinetics were studied by fitting the experimental data with linear equations of pseudo-first-order and pseudo-second-order kinetic models (Figure 6C,D and Figure S3). Studying the kinetics of an adsorption process is important to determine the adsorption rate and mechanism. The parameters for both models and their linear correlation coefficients for all the adsorbents are presented in Table 2. The linear correlation coefficient values (R^2^) were always higher (closer to the unit) for the pseudo-second-order model, and the calculated equilibrium adsorption capacities from the pseudo-second-order model were closer to the experimental values (*Q_e_*, Exp.). These indicate that the adsorption of Cr(VI) ions onto neat PPy, PPy/TA, PPy/NFC, and PPy/NFC/TA aerogels follows pseudo-second-order kinetics and implies that the rate-limiting step of Cr(VI) adsorption is a consequence of the chemisorption process. The chemisorption mechanism can be due to the complexation between Cr(VI) ions and TA functional groups, and ion exchange with Cl^−^ ions (dopant) on PPy chains (Figure S4).

**Figure 7:**
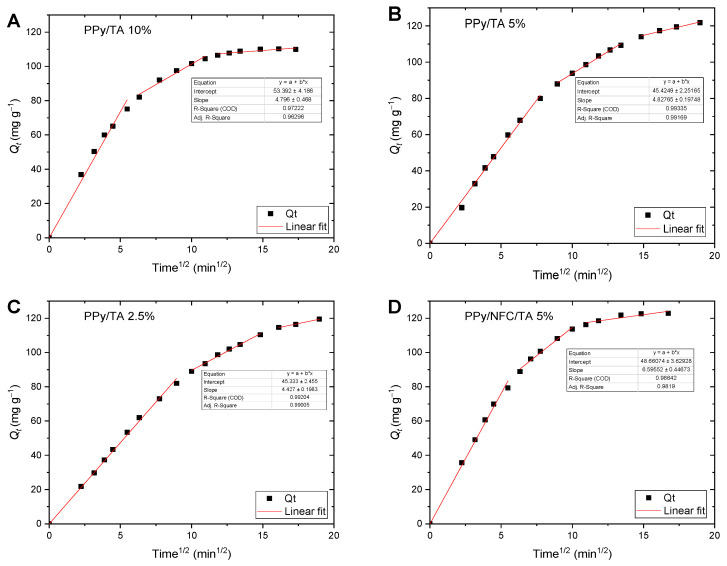

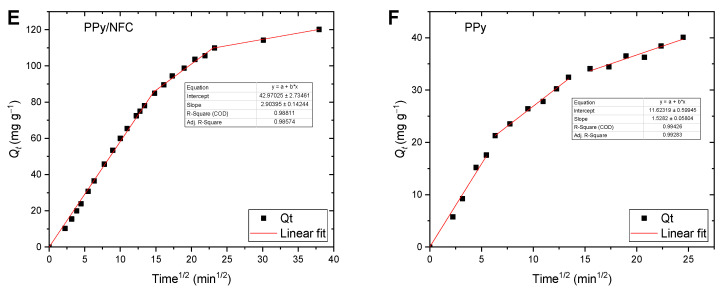
Intraparticle diffusion modeling of Cr(VI) ions adsorption onto PPy/TA aerogels prepared with 10 (**A**), 5.0 (**B**), and 2.5 (**C**) mole% of TA, PPy/NFC/TA 5.0% (**D**), PPy/NFC (**E**), and neat PPy powder (**F**).

The Weber–Morris plots (intraparticle diffusion model) of the adsorption capacity of Cr(VI) ions onto PPy/TA aerogels with different TA concentrations, PPy/NFC/TA, PPy/NFC aerogels and neat PPy vs. *t*^1/2^ are shown in Figure 7. These intraparticle diffusion plots predict that the adsorption of Cr(VI) ions occurs in multiple stages and imply that surface adsorption is not the only mechanism that controls the adsorption process [38]. At the same time, this implies that intraparticle diffusion plays an important role in the adsorption process without being the sole rate-determining parameter. The first adsorption step happens by surface adsorption, the second step is due to the gradual diffusion stage, where intraparticle diffusion is the rate-limiting parameter, and the third plateau region represents the adsorption equilibrium [39]. The slope and the intercept of the second linear section of the plots represent the intraparticle diffusion constants *k_i_* and *c_i_*, respectively. Intraparticle diffusion parameters are listed in Table 2. The larger the c_i_ value is, the lower is the effect of diffusion as a rate-limiting step.

### 2.4. Equilibrium Adsorption Isotherms

**Figure 8:**
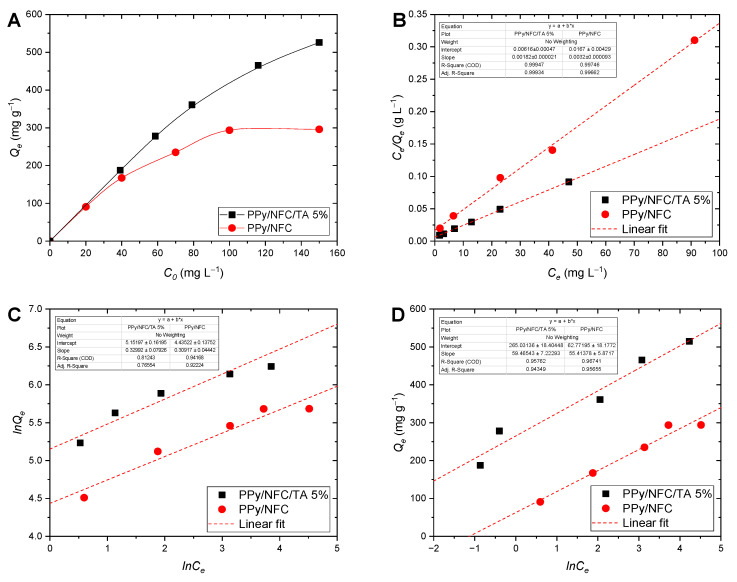
The relation between initial concentration and the adsorption capacity (**A**), Langmuir isotherm (**B**), Freundlich isotherm (**C**), and Temkin isotherm (**D**) modeling.

The maximum adsorption capacity and the adsorption mechanism were estimated by studying the equilibrium isotherms. PPy/NFC/TA 5% was chosen for the equilibrium adsorption isotherms study due to its mechanical stability and best adsorption kinetics. In addition, no TA was detected to leach from this aerogel. The equilibrium isotherm models of Langmuir, Freundlich, and Temkin were fitted with the experimental results of PPy/NFC/TA 5% and compared with PPy/NFC aerogel prepared without TA (Figure 8).

The experimental results fit perfectly with the Langmuir isotherm model with the highest R^2^ values (0.999). This indicate that the adsorption of Cr(VI) ions onto the PPy aerogels follows the Langmuir model better than other models. The Langmuir isotherm assumes the adsorption of a homogenous monolayer of the adsorbate (Cr(VI) ions) onto the surface of the adsorbents (aerogels). The Freundlich isotherm describes the adsorption of heterogeneous multilayers, while the Temkin isotherm describes the heat of adsorption due to the adsorbate (metal ions) and the adsorbent (PPy aerogels) interactions. The calculated parameters of isotherms and the linear coefficient (R^2^) are listed in Table 3. The calculated Langmuir *Q_max_* of 549.5 and 312.5 mg g^−1^ for PPy/NFC/TA 5% and PPy/NFC, respectively, correlates well with the experimentally calculated values (Table 3). Due to the addition of TA to the PPy/NFC, the adsorption capacity has significantly increased, to be almost doubled. These results indicate the high affinity of PPy/NFC/TA 5% for Cr(VI) compared with most of the previously reported PPy-based materials. Free-standing bacterial cellulose/PPy sheets revealed an adsorption capacity of 294.1 mg g^−1^ at pH 3 [10]. PPy–polyvinyl alcohol showed an adsorption capacity of 497.5 mg g^−1^ due to its high porosity [17]. PPy/dolomite composite has a maximum adsorption capacity of 406.5 mg g^−1^ at 50 °C [40]. PPy-modified eggplant aerogel possessed 27.83 mg g^−1^ adsorption capacity [41]. Ultralight and super-elastic aerogel of PPy/cellulose acetate nanofibers showed a maximum adsorption capacity of 322.58 mg/g at 45 °C [42].

### 2.5. Partition Coefficient

**Figure 9:**
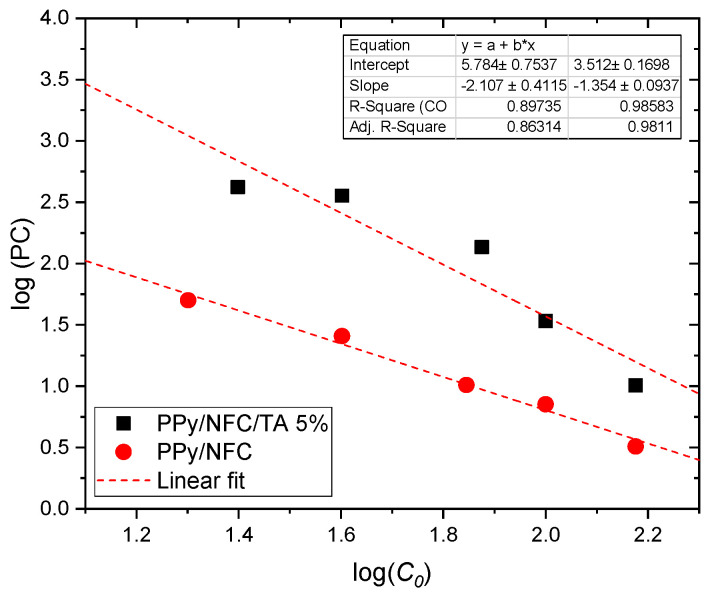
Regression plots for the adsorption performance of PPy/NFC and PPy/NFC/TA 5% aerogels toward Cr(VI) ions using the partition coefficient as a function of initial Cr(VI) concentration.

For a better understanding of the adsorption performances of PPy/NFC compared with PPy/NFC/TA 5% aerogels, their partition coefficient (PC; L g^−1^) was compared. Unlike the removal efficiency and adsorption capacity, PC does not depend on the operating conditions. PC indicates the real adsorption performance and helps to understand the affinity of a Cr(VI) for the aerogel compared with the aqueous phase. PC is the ratio of adsorbate concentration in the solid phase to the concentration in the liquid phase at equilibrium (Equation (9)) [43]. Figure 9 represents the calculated PC of PPy/NFC aerogel prepared in the absence and presence of TA as a function of initial Cr(VI) concentrations. 

The results show that PPy/NFC aerogel prepared in the presence of TA has higher PC than PPy/NFC prepared without TA at different initial concentrations of Cr(VI) ions. This means that PPy/NFC/TA has a higher affinity for Cr(VI) anionic species in the solution. Additionally, it was observed that, by increasing the Cr(VI) initial concentration, the PC decreases due to the occupation of active sites on the aerogels.

## 3. Conclusions

PPy/TA aerogels were prepared through a single-step method with different TA concentrations. The addition of NFC enhanced the mechanical stability of the PPy/TA aerogel. Conductivity was found to decrease when increasing the TA content in the aerogel. Conductivities of 9.6 ± 1.7 and 0.07 ± 0.01 S cm^−1^ were achieved for PPy/TA prepared with 2.5% and 10% of TA, respectively. FTIR and Raman spectroscopies revealed the electrostatic interaction between TA and PPy chains. PPy aerogels were utilized as adsorbents for removing Cr(VI) ions from aqueous solutions, and their adsorption efficiency was compared. For all the PPy aerogels, the adsorption process followed pseudo-second-order kinetics, indicating a chemisorption process. Due to the addition of TA, the maximum adsorption capacity of PPy/NFC aerogels increased from 312.5 mg g^−1^ (for PPy/NFC aerogel) to 549.5 mg g^−1^ (for PPy/NFC/TA 5%). These findings reveal the promising nature of such aerogels in environmental applications as efficient adsorbents in water treatment.

## 4. Materials and Methods

### 4.1. Chemicals and Reagents

Pyrrole, iron(III) chloride hexahydrate and tannic acid were obtained from Sigma-Aldrich (China), and NFC (3 wt% aqueous suspension) was obtained from the University of Maine (Orono, ME, USA). All chemicals were used as received without any further purifications.

### 4.2. Preparation of Polypyrrole Aerogels

A series of PPy/TA cryogels were prepared by varying the TA concentration. Pyrrole (0.2 M, 0.671 g) was mixed with different concentrations of TA solution (5 × 10^−3^ M, 0.01 M, and 0.02 M, 25 mL) in Milli-Q water to obtain mole ratios of 2.5, 5, and 10% of TA to pyrrole. An oxidant mixture of iron(III) chloride (0.25 M, 3.379 g) and ammonium persulfate (0.12 M, 1.26 g) was dissolved in a 25 mL measuring flask. The separate solutions were previously cooled down to near frozen conditions (0 °C). The monomer/TA and oxidant solutions were then mixed and immediately frozen in an ethanol/dry ice bath (−78 °C) for 30 min before being transferred to a freezer (−24 °C). In the same way, PPy/NFC/TA cryogels were prepared by mixing 0.7 wt% NFC with TA (0.01 M, 5 mole%) and pyrrole (0.2 M), then adding the oxidant. The cryogels were kept in the freezer (−24 °C) for one week, then thawed at room temperature, and rinsed in a large amount of 0.2 M HCl to remove any byproducts. The corresponding aerogels were obtained by freeze-drying the cryogels (GREGOR Instruments, L4-110 freeze-drier, Sázava, Czech Republic). Globular PPy was prepared under frozen conditions, as described elsewhere [33]. Pyrrole solution (0.2 M, 25 mL) was mixed with iron(III) chloride solution (0.25 M, 25 mL) in a water/ethylene glycol bath at −24 °C. PPy powder was collected after thawing by filtration, washed with 0.2 M HCl solution, then with ethanol, and dried over silica gel until constant weights were obtained.

### 4.3. Characterization

The morphology of the aerogels was investigated with a MAIA3 TESCAN scanning electron microscope (Brno, Czech Republic).

The DC conductivity of the aerogels was measured on pressed pellets by the van der Pauw method at 22 ± 1 °C and relative humidity at 38 ± 5%. About 120 mg of the aerogels was ground and pressed into pellets (13 mm diameter, <1 mm thickness) using Trystom H-62 manual hydraulic press (Olomouc, Czech Republic) at 70 kN. A programmed potential was applied using a Keithley 230 voltage source, the current was measured using a Keithley 617 electrometer, and the voltage drop across the sample was measured with another Keithley 617 electrometer (Cleveland, Ohio, USA). The conductivity was calculated from the current–voltage plot slope. Three measurements for each sample in reciprocal directions were performed, and the average values were calculated.

The FTIR spectra of the aerogels were recorded with a Thermo Nicolet NEXUS 870 FTIR spectrometer with a DTGS TEC detector (Madison, WI, USA) in the wavenumber range of 400–4000 cm^−1^.

Raman spectra of aerogels excited with a NIR diode laser (emitting at 785 nm) were recorded with a Renishaw InVia Reflex Raman microspectrometer (Wotton-under-Edge, UK). The scattered light was analyzed using a spectrograph with holographic gratings of 1200 lines mm^−1^ and 1800 lines mm^−1^. A research-grade Leica DM LM microscope was used to focus the laser beam. A Peltier-cooled CCD detector (576 × 384 pixels) registered the dispersed light.

Thermal gravimetric analysis of the aerogels was performed in air at a heating rate of 10 °C min^−1^ up to 800 °C using a PerkinElmer Pyris 1 Thermogravimetric Analyzer (Fremont, CA, USA).

### 4.4. Removal of Chromium Ions

A stock solution of Cr(VI) was prepared in Milli-Q water by dissolving 353.61 mg K_2_Cr_2_O_7_ powder in a 250 mL measuring flask to prepare a Cr(VI) concentration of 500 mg L^−1^, which can be further diluted to lower concentrations. For a typical batch adsorption experiment, 0.05 g of the aerogels was mixed with aqueous solutions of Cr(VI) solutions (25 mL, 25 mg L^−1^) at pH 2, room temperature (22 ± 1 °C), 150 rpm shaking rate, and dark conditions. The concentration of Cr(VI) was followed by measuring the UV–visible spectra using a Thermo Scientific Evolution 220 UV–visible spectrophotometer (Waltham, MA, USA) over time.

Adsorption capacity (*Q_t_*, mg g^−1^) at time *t* (min) and removal efficiency (*RE*, %) were calculated as follows:

(1) $$Q_{t}=\frac{\left(C_{i}-C_{t}\right)}{m}\times V$$

(2) $$\mathrm{RE}=\frac{\left(C_{i}-C_{e}\right)}{C_{i}}\times 100$$

where *C_i_* (mg L^−1^) is the initial concentration of Cr(VI), *C_t_* (mg L^−1^) is the concentration of Cr(VI) at time *t* (min), *C_e_* (mg L^−1^) is the concentration of Cr(VI) in the solution at equilibrium, *V* (L) is the volume of Cr(VI) solution, and *m* (g) is the mass of the adsorbent.

The adsorption kinetics of Cr(VI) ions were studied within 24 h using the linearized models of pseudo-first-order (Equation (3)) and pseudo-second-order models (Equation (4)), and intraparticle diffusion (Equation (5)) as follows:

(3) $$ln(Q_{e}-Q_{t})={\mathrm{lnQ}}_{e}-k_{1}t$$

(4) $$t/Q_{t}=1/k_{2}Q_{e}^{2}+t/Q_{e}$$

(5) $$Q_{t}=k_{i}t^{1/2}+c_{i}$$

where *Q_e_* and *Q_t_* (mg g^−1^) are the capacities of Cr(VI) ions at equilibrium and time *t* (min), respectively, *k*_1_ (min^−1^) is the pseudo-first-order rate constant, *k*_2_ (g mg^−1^ min^−1^) is the pseudo-second-order rate constant, *k_i_* (mg g^−1^ min^−1/2^) is the intraparticle diffusion rate constant, and *c*_i_ (mg g^−1^) is the intraparticle diffusion constant.

The equilibrium isotherms were determined for the selected adsorbents in a batch mode by mixing 0.005 g of the aerogels with varied concentrations of Cr(VI) solutions (25 mL) within the range 25–150 mg L^−1^, at pH 2, room temperature, and 200 rpm stirring. The equilibrium point was taken after 48 h. The experimental data were fitted against Langmuir (Equation (6)), Freundlich (Equation (7)), and Temkin (Equation (8)) isotherm models as follows:

(6) $$C_{e}/Q_{e}=1/Q_{\max}K_{L}+C_{e}/Q_{\max}$$

(7) $${\mathrm{lnQ}}_{e}={\mathrm{lnK}}_{F}+\frac{1}{n}{\mathrm{lnC}}_{e}$$

(8) $$Q_{e}=B{\ln K}_{T}+B\ln C_{e}$$

where *Q_max_* (mg g^−1^) is the Langmuir maximum adsorption capacity at saturation, *K_L_* (L mg^−1^) is the Langmuir constant that describes the affinity between the adsorbate and the adsorbent; the higher the Langmuir constant value is, the more stable is the adsorption process is. *K_F_* (mg g^−1^) is the Freundlich constant that describes the adsorption capacity, *K_T_* (L g^−1^) is the Temkin isotherm constant that corresponds to the maximum binding energy, and *n* is a Freundlich constant related to the efficiency of the adsorption; adsorption is favorable when 0.1 < 1/*n* < 1 (the bigger the 1/*n* is, the higher is the favorability). *B* is a Temkin constant related to the heat of adsorption.

The best model fitting and validity of the kinetic and isotherm models were determined by the linear correlation coefficient (R^2^).

The partition coefficient of Cr(VI) adsorption onto the aerogels measures the distribution of adsorbate between the solution and adsorbents. It is calculated as follows:

(9) $$\mathrm{PC}=Q_{e}/C_{e}$$

where *Q_e_* (mg g^−1^) and *C_e_* (mg L^−1^) are adsorption capacity and concentration of the Cr(VI) at equilibrium, respectively.

## Figures and Tables

**Table 1:** DC conductivity of PPy and PPy/TA aerogels.

Sample	Conductivity, S cm^−1^
PPy	23.7 *
PPy/TA (2.5%)	9.6 ± 1.7
PPy/TA (5.0%)	0.95 ± 0.13
PPy/TA (10%)	0.07 ± 0.01
PPy/NFC/TA (5.0%)	0.37 ± 0.017

* Reference no. [33,34].

**Table 2:** Adsorption kinetics parameters.

Adsorbent	*Q_e_*, Exp. Mg g^−1^	Pseudo-First-Order			Pseudo-Second-Order			Intraparticle Diffusion		
		*Q_e_*, mg g^−1^	*k_1_*, min^−1^	R^2^	*Q_e_*, mg g^−1^	*k_2_*, gmg^−1^ min^−1^	R^2^	*c_i_*, mg g^−1^	*k_i_*, mgg^−1^ min^−1/2^	R^2^
PPy/TA 10%	110.2	69.5	0.019	0.985	111.2	9.8 × 10^−4^	0.999	53.40	4.79	0.972
PPy/TA 5%	124.1	95.6	0.011	0.994	133.3	2.0 × 10^−4^	0.999	45.43	4.83	0.993
PPy/NFC/TA 5%	123.5	89.4	0.021	0.993	132.4	4.3 × 10^−4^	0.999	48.66	6.59	0.986
PPy/TA 2.5%	122.2	97.4	0.010	0.993	125.6	2.3 × 10^−4^	0.999	45.33	4.43	0.992
PPy/NFC	120.1	100.3	0.004	0.986	127.7	7.9 × 10^−5^	0.998	42.90	2.90	0.988
PPy	46.61	24.17	0.0034	0.982	45.45	3.3 × 10^−4^	0.997	11.62	1.53	0.994

**Table 3:** Equilibrium isotherm parameters.

Aerogel	*Q_max_*, Exp., mg g^−1^	Langmuir			Freundlich			Temkin		
		*Q_max_*, mg g^−1^	*K_L_*, L mg^−1^	R^2^	*K_F_*, mg g^−1^	1/*n*	R^2^	*K_T_*, L g^−1^	*B*	R^2^
PPy/NFC/TA 5%	515.6	549.5	0.296	0.999	172.78	0.330	0.812	4.46	59.47	0.957
PPy/NFC	296.0	312.5	0.192	0.997	84.35	0.309	0.922	1.13	55.41	0.967

## Data Availability

Data are available on request.

## Supplementary Materials

The following supporting information can be downloaded at: https://www.mdpi.com/article/10.3390/gels10070415/s1. Figure S1: SEM micrograph of neat PPy prepared under frozen conditions at −24°C; Figure S2: UV-visible spectra of TA solution and Cr(VI) solutions after mixing with PPy/TA 10%, PPy/TA 5% and PPy/TA 2.5%, PPy/NFC/TA 5% for 24 h; Figure S3: UV-visible spectra of Cr(VI) solution over time while adsorbing onto neat PPy; Figure S4: Adsorption mechanism of Cr(VI) ions onto PPy/TA aerogels.
