# Supplementary material for: Aerogels of Polypyrrole/Tannic Acid with Nanofibrillated Cellulose for the Removal of Hexavalent Chromium Ions
Source: Gels. 2024 Jun 22;10(7):415. doi: 10.3390/gels10070415 (PMC11275629; doi:10.3390/gels10070415)
Supplement: Supplementary file 1 [file gels-10-00415-s001.zip › gels-3056994-supplementary.pdf]

Supplementary Materials

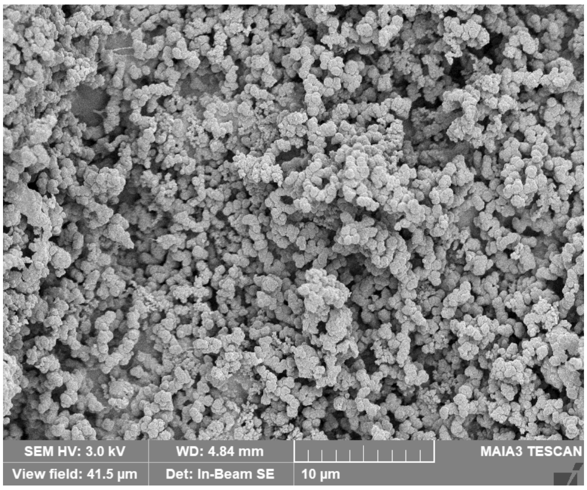

Figure S1. SEM micrograph of neat PPy prepared under frozen conditions at −24°C.

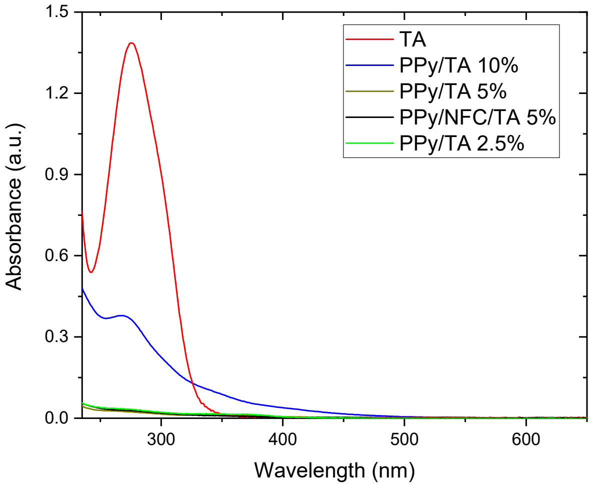

Figure S2. UV-visible spectra of TA solution and Cr(VI) solutions after mixing with PPy/TA 10%, PPy/TA 5% and PPy/TA 2.5%, PPy/NFC/TA 5% for 24 h.

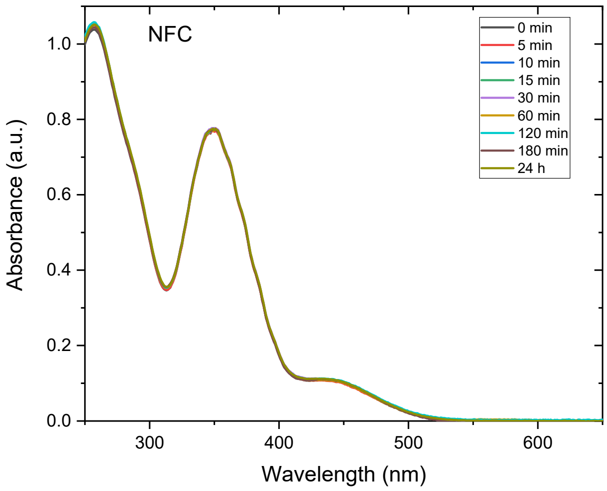

Figure S3. UV-visible spectra of Cr(VI) solution over time while adsorbing onto neat PPy.

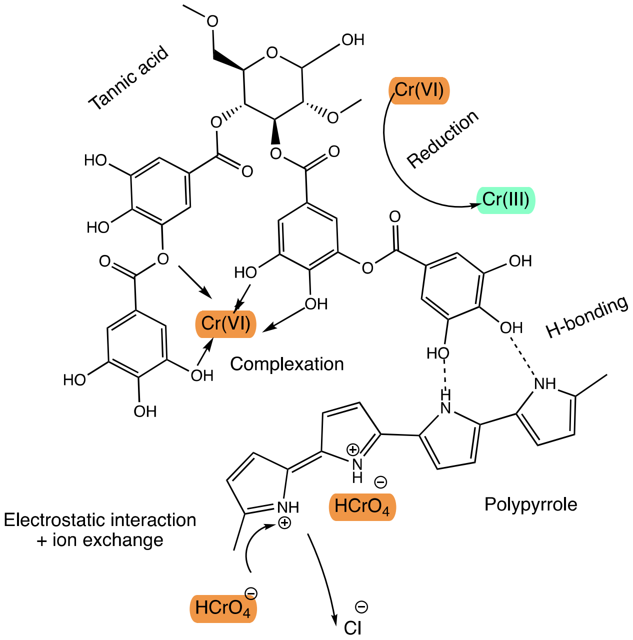

Figure S4. Adsorption mechanism of Cr(VI) ions onto PPy/TA aerogels.
